# The Confluence Model of Sexual Aggression: The Role of Pornography as a Secondary Risk Factor

**DOI:** 10.5964/sotrap.13005

**Published:** 2023-11-24

**Authors:** Sundus Saqib, Myles Davidson

**Affiliations:** 1 Department of Psychology, Saint Mary’s University, Halifax, NS, Canada; Centre for Criminology (Kriminologische Zentralstelle – KrimZ), Wiesbaden, Germany

**Keywords:** pornography, pornography use motives, Confluence Model, sexual aggression

## Abstract

The Confluence Model (CM) highlights the role of Hostile Masculinity (HM) and Impersonal Sex (IS) as primary risk factors for sexual aggression. According to the CM, pornography is a secondary risk factor for sexual aggression among men who are high in HM and IS. The role of pornography consumption in the CM has produced divergent findings; however, a subset of men use pornography for mood management (to cope with negative emotions). These men are at risk of developing problematic pornography use, suggesting a need to consider motives for pornography use in the context of the CM. The present study tested the CM with a focus on the role of pornography as a mood management motive in a sample of males (n = 412). The central components of the CM (HM and IS) were associated with sexual aggression. There was also an independent association between the mood management motive of watching pornography and sexual aggression, suggesting that the mood management motive of watching pornography may be an independent risk factor for sexual aggression. In contrast to our hypothesis, the mood management motive did not moderate the association between the primary risk factors (HM, IS) and sexual aggression. The findings of this study replicate past findings and build on the literature by suggesting that pornography use as mood management is predictive of sexual aggression.

## Abstract (plain language summary)

**Background:**

The Confluence Model (CM) is a framework of sexual aggression developed in 1986 and is used to predict the likelihood of sexual aggression perpetration in men. Hostile Masculinity (HM) and Impersonal Sexuality (IS) are the core components of this model and serve as primary risk factors for sexual aggression. It is thought that if men possess these primary risk factors, pornography consumption becomes a secondary risk factor. Despite this, it is not yet known the role pornography plays in the CM. This is the first study to test pornography use as a secondary risk factor in the CM using a reliable and valid scale.

**Why was this study done?:**

We sought to determine whether the risk factors proposed to predict sexual aggression three decades ago are still relevant to measuring sexual aggression in the current day. Moreover, we wanted to test whether pornography use plays a role in sexual aggression for men who possess the primary risk factors. Instead of measuring general pornography use, we were interested in investigating if using pornography to manage one’s mood (i.e., watching pornography to suppress negative emotions) would serve as a secondary risk factor for sexual aggression.

**What did the researchers do and find?:**

We originally surveyed 616 participants and conducted statistical analyses on 412 participants. To participate, individuals had to be emerging young male adults (aged between 17-25) who experienced a sexual attraction to women and resided in North America at the time of the study. Participants were presented with questions related to sexual aggression, hostile attitudes toward women, impersonal sexual tendencies, pornography consumption, and motivations for watching pornography. Participants who scored high on the core components of the CM reported being sexually aggressive. Additionally, participants who watched pornography to manage their mood also reported sexual aggression. However, watching pornography as a motive to manage mood did not impact the individual relationship between the core components of the CM and sexual aggression.

**What do these findings mean?:**

The findings from this study are consistent with past research testing the CM. Understanding the core components of the CM is still relevant in predicting the likelihood of sexual aggression today. Our study sheds additional light on the CM by demonstrating the utility of using an established scale to measure pornography use in this context. Furthermore, it demonstrates that motivations for using pornography may lead to sexual aggression. Overall, our findings can be helpful for future researchers investigating the role pornography plays in both the CM and sexually aggressive behaviour, as well as for clinicians who work with pornography users in understanding the motivations behind their use of pornography.

## Highlights

- Some men who report being sexually aggressive may use pornography for mood management.
- Using pornography for mood management does not moderate the relationships in the CM.
- There is a relationship between using pornography for mood management and sexual aggression.
- This study is the first to measure pornography use alongside the CM using a validated scale.
- Using pornography for mood management should be a treatment target for sexually aggressive men.

The Confluence Model (CM) of Sexual Aggression is a well-established theoretical model designed to understand specific characteristics that can predict the likelihood of men’s sexual aggression perpetration against women (Malamuth, 1998). The CM is one of the most widely tested theoretical models in studies of sexual aggression and has received significant empirical support (Kohut et al., 2021; Malamuth et al., 1995; Malamuth & Hald, 2016). The CM identifies the characteristics of men in the general population that increase their risk of both general and sexually aggressive behaviours. Further, the CM also considers factors that are primary to the development of sexual aggressive tendencies in addition to more secondary factors (Malamuth, 2018).

The CM presents a reasonable rationale that links pornography usage to sexual aggression, which was a later addition to the model (Malamuth, 2018; Malamuth et al., 2000; Malamuth et al., 2021; Vega & Malamuth, 2007). There have been many debates on the association between pornography use and sexual aggression and findings have been mixed (e.g., Bauserman, 1996; Dawson et al., 2019; Ferguson & Hartley, 2022; Kingston et al., 2008; Kohut et al., 2021; Wright et al., 2016; Yang & Youn, 2012). Therefore, whether pornography consumption is related to sexual aggression continues to be debated in the literature. The purpose of this study was to test the CM of sexual aggression with a specific focus on the role of pornography. The study extends the research literature in this area by considering the more specific factor of pornography use motives in sexual aggression.

## Overview of the Confluence Model of Sexual Aggression

The CM was initially developed by Malamuth (1986) and has been further developed since that time (Malamuth et al., 1991; Malamuth et al., 1995; Malamuth et al., 2021). The CM emphasizes the role of specific primary and secondary risk factors when exploring what impacts men’s perpetration of sexual aggression against women (Malamuth & Hald, 2016). The primary risk factors are assumed to be more formative in the development of risk for sexual aggression and include general characteristics such as stable personality features (e.g., psychopathic tendencies and antisociality) resulting from previous traumatic experiences (Dawson et al., 2019; Malamuth, 2018). The primary risk factors are also specific to the risk for sexual aggression (e.g., attitudes supporting violence towards women, impersonal sexual orientation). The CM posits that a man’s likelihood of perpetrating sexual aggression is best understood by the confluence of two largely independent but related constellations of risk factors: Hostile Masculinity (HM), or a distrustful and hostile attitude toward women; and Impersonal Sexuality (IS), or a non-committal perspective of sex (Malamuth, 2018; Malamuth et al., 1995; Malamuth et al., 2021). In other words, it is assumed that if a man is relatively high on the factors contained within these constellations (HM and IS), then he is at risk for being sexually aggressive (Malamuth et al., 1991; Malamuth et al., 1995).

### Hostile Masculinity (HM)

HM is described as a “narcissistic, insecure, defensive, hypersensitive, and hostile-distrustful orientation” towards women (Malamuth & Hald, 2016, p. 54). A man who is high in HM is considered to have a hostile orientation typically toward women, and satisfaction is achieved through dominating and manipulating women (Malamuth, 1998; Malamuth & Hald, 2016; Malamuth et al., 1995). Further, a man high in HM is afraid of rejection and is anxious about relationships with women, which comes from a sense of insecurity and defensiveness (Malamuth et al., 1995). Thus, the use of coercion against women derives from the fear of being rejected. The characteristics representative of HM extend beyond a misogynous hostile-distrustful orientation towards women and a sexual desire to dominate women to also include the acceptance of rape myths (Baer et al., 2015; Huntington et al., 2020; Kingston et al., 2008; Kohut et al., 2021; Malamuth & Hald, 2016; Malamuth et al., 1995).

### Impersonal Sexuality (IS)

IS [also occasionally referred to as sexual promiscuity (SP)] is represented by a “promiscuous detached orientation toward sexual relations” (Malamuth & Hald, 2016, p. 54). Malamuth et al. (1991) and Malamuth et al. (1995) originally identified both impersonal sex (IS) and sexual promiscuity (SP) as separate, but related, independent constructs. Another study by Malamuth (1996) reported that impersonal sex is also referred to as sexual promiscuity, despite having previously recognized them as independent constructs. Therefore, the IS variable of the CM suffers from not having a strong conceptual definition which also creates challenges in the creation of a reliable and valid measure of IS.

Research suggests that the IS pathway refers to a developmental history of growing up in a “harsh” environment (e.g., being surrounded by violence or abuse) that leads to antisocial tendencies and a “detached” view of relationships (Malamuth, 1998; Malamuth, 2018; Malamuth & Hald, 2016; Malamuth et al., 1991; Malamuth et al., 1995; Malamuth et al., 2021). Sex is considered impersonal when commitment, emotional intimacy, and exclusivity are not present or not valued (Malamuth & Hald, 2016; Malamuth et al., 2012). In other words, IS refers to a game-playing orientation toward sexual activity and is described as the willingness to engage in sexual acts without the presence of commitment (Malamuth et al., 1995). Thus, people high in IS favor non-committed casual sexual relationships over long-term relationships (Abbey et al., 2011; Malamuth et al., 1995). Other aspects of the IS construct include having multiple dating partners and being sexually active at a younger age (Huntington et al., 2020).

## Research Support for the CM

The CM has received strong empirical support due to its ability to predict sexual aggression (Malamuth, 1998; Malamuth et al., 1995; Malamuth et al., 2021). Initially, a cross-sectional study by Malamuth (1986) examined self-reported sexual aggression in a sample of 155 males. The results of the study revealed that dominance as a motive for sex, hostility toward women, attitudes supporting violence, and arousal to sexual aggression were significantly related to self-reported sexual aggression; however, the interaction of these variables resulted in higher levels of sexual aggression. Another study by Malamuth et al. (1991) analyzed characteristics of aggressors against women in 2,652 college men. They found that factors such as growing up in a hostile home environment, general delinquency, sexual promiscuity, and having a hostile personality were related to sexual aggression against women. Moreover, they concluded that the confluence and interaction of Hostile Masculinity (HM) and Impersonal Sexuality (IS) produced the highest levels of sexual aggression. Thus, Malamuth labeled the model combining these constellations as “The Confluence Model”.

Additionally, Malamuth et al. (1995) posited that a good approach to understanding the risk of sexual aggression perpetration is by examining the confluence of the two paths. In other words, the CM of sexual aggression was designed considering developmental, personality, and behavioral elements which were initially associated with a specific behavior (i.e., HM and IS) among male perpetrators in the general population (Malamuth & Hald, 2016). Nonetheless, there have been various debates on whether one or both pathways may be more strongly associated with sexual aggression. Hence, reviewing various empirical studies of the CM can help better understand the debate.

An examination of longitudinal studies is key to understanding the CM of sexual aggression. An earlier longitudinal study by Malamuth et al. (1995) followed up with a sample of men 10 years after initially studying them when they were adolescents. The researchers tested some components of the CM to predict which men were likely to engage in sexual aggression and other types of conflict with women. The results of this study revealed that sexual aggression is best understood by the combination of relatively high levels of HM and SP (IS); however, they suggested that when men with higher levels of HM choose to engage in casual sex (IS), it is more likely to be coercive. Further, a recent longitudinal study by Kohut et al. (2021) tested some components of the CM by using two independent longitudinal panel samples (Rijeka vs Zagreb) of male Croatian adolescents. They observed the link between HM and self-reported sexual aggression in both panels; however, the results also revealed that there were no significant effects of IS in both panels.

Although different studies have revealed diverse outcomes regarding the two paths of the CM, the key feature of the CM is that while both HM and IS can be individually related to sexual aggression, the interaction of these two factors is the most important element of the model’s predictive utility (Malamuth et al., 1991; Malamuth et al., 1995). In addition to these primary risk factors, the CM acknowledges that the secondary risk factors may interact with the primary risk factors. Of greatest relevance to the present study, the CM identifies pornography as a proximate, secondary factor that can interact with the primary risk factors of the CM (Malamuth, 2018; Malamuth et al., 2000).

## The Role of Pornography as a Secondary Risk Factor in the Confluence Model

Due to numerous studies supporting the use of the CM in predicting the risk of sexual aggression perpetration (Malamuth et al., 1991; Malamuth et al., 1995; Malamuth et al., 2021), several constructs have been added to the model to increase its predictive utility. Malamuth and Hald (2016) proposed that the reason the CM is a useful model for examining other risk factors associated with sexual aggression is that the model has already identified reliable correlates of sexual aggression. Thus, the CM also considers the role of secondary risk factors which include general hostile personality traits, low nurturance, substance use, and pornography consumption (Malamuth, 2018; Malamuth & Hald, 2016; Malamuth et al., 2021).

The CM first incorporated pornography consumption about two decades ago (Malamuth et al., 2000). Although most research has focused on the core components of the CM (HM and IS), some studies support the notion that pornography use may be a secondary risk factor for sexual aggression in men who already scored high in both HM and IS (Malamuth et al., 2000). This is because secondary risk factors are seen as disinhibitory/inhibitory factors with substantial influence when the primary factors are high (Malamuth et al., 2021). Therefore, if a man is assumed to have high levels of primary risk factors, he may also develop high levels of secondary risk factors. In 2018, Malamuth expanded the framework to include child pornography as a risk factor for sexual aggression against children. Despite this, the CM remains primarily used for sexual aggression against adult women. Taking this into consideration, however, conclusions from the CM can be applied not only to adult pornography but to the impacts of child pornography as well (Malamuth, 2018).

## Pornography and Sexual Aggression

Before discussing the research on the role of pornography within the context of the CM, it is crucial to provide a brief discussion on the independent association between pornography consumption and sexual aggression. The possible impact of pornography on men’s sexual aggression against women is a topic of substantial interest to the public and has been the subject of many studies (Bauserman, 1996; Dawson et al., 2019; Malamuth et al., 2000; Vega & Malamuth, 2007; Wright et al., 2016; Yang & Youn, 2012). Nonetheless, researchers have not come to an agreement about whether there is an association or not (Ferguson & Hartley, 2022; Wright et al., 2016). Given the discrepancies in individual studies, meta-analyses can provide helpful clarification on the relationship between pornography use and sexual aggression.

A meta-analysis conducted by Wright et al. (2016) combined cross-sectional and longitudinal studies from different countries (conducted between 1994 to 2011) to examine the association between pornography consumption and sexual aggression. Their results revealed that there was a small positive correlation between pornography consumption and sexually aggressive behavior. They also discovered that associations between pornography use were stronger for verbal acts of sexual aggression compared to physical sexual aggression. Additionally, it was revealed that this association was stronger in cross-sectional studies compared to methodologically stronger longitudinal studies.

A subsequent meta-analysis by Ferguson and Hartley (2022) of correlational, experimental, and population studies (conducted between the 1970s to 2020) also examined the influence of pornography on sexual aggression. This was the first meta-analysis that considered each study’s methodological issues which could influence effect sizes. Overall, conclusions from their study revealed largely non-significant effects of non-violent pornography on sexual aggression. Regarding violent pornography, both correlational and experimental studies demonstrated a small effect size. Interestingly, the population studies revealed an inverse relationship between pornography consumption and sexual aggression (e.g., higher availability of pornography was associated with reduced sexual aggression levels). However, their sample sizes consisted of European and American majority populations which are not globally representative.

Given the opposing findings, researchers have not come to a consensus about pornography being a reliable correlate of sexually aggressive behavior. Thus, examining the role of primary risk factors for sexual aggression in tandem with this secondary risk factor may help shed light on the role pornography has on men’s perpetration of sexual aggression against women.

## Empirical Support of Pornography in the Confluence Model

Several researchers have tested the primary risk factors of the CM in confluence with the secondary risk factor of pornography consumption. For instance, the first paper testing the CM and the role of pornography as a secondary risk factor was conducted by Malamuth et al. (2000). This cross-sectional study tested the CM in a representative sample of 2,927 men with a mean age of 21. The researchers first categorized their participants into “low”, “moderate”, and “high” risk groups depending on their HM and IS scores. They then considered the risk levels to measure the association between pornography consumption and sexual aggression. Their results suggested an interaction effect for males in their sample who achieved a high-risk score (high scores in both HM and IS) and consumed high amounts of pornography. Thus, pornography consumption tends to be highest in men who are at high-risk for sexually aggressive behaviour.

Vega and Malamuth (2007) replicated Malamuth et al.’s (2000) study and tested the CM in a sample of 102 male college students in Los Angeles. They also concluded that men who were categorized as high-risk for sexual aggression consumed high amounts of pornography. Thus, men who score high on the CM risk factors may be attracted to images in pornography that reinforce and increase the likelihood of their impersonal and hostile orientation to sexuality, thereby increasing their overall risk for sexual aggression (Malamuth, 2018; Malamuth et al., 2000).

In contrast, a recent longitudinal study by Kohut et al. (2021) examining the role of pornography in the CM revealed different results. Unlike previous research (Malamuth, 2018; Malamuth et al., 2000; Malamuth et al., 2012; Vega & Malamuth, 2007), results from Kohut et al. (2021) revealed that pornography use did not affect sexual aggression regardless of participants’ risk level. Thus, pornography did not moderate the association between the core components of the CM and sexual aggression. Kohut et al. (2021) acknowledged that this finding may be due to cultural differences between the previous North American samples and the Croatian panel samples.

## Incorporating Pornography Use Motives to the Confluence Model

Although pornography consumption is an important consideration in the context of the CM, little focus is given to the reason people use pornographic material online (Paul & Shim, 2008). Not many studies have specifically attempted to understand people’s needs or motivations for Internet pornography usage. Thus, the present study aims to consider a man’s motives for pornography usage and how it is associated with the CM of sexual aggression. Paul and Shim (2008) emphasized that it is crucial to understand the motives for watching pornography because people’s selection and use of a particular medium are strongly associated with their motivations to fulfill and satisfy specific needs.

Paul and Shim (2008) proposed that pornography consumption can be clustered into four underlying motives: relationship motives, fantasy motives, habitual use motives, and mood management motives. The relationship motive indicates that people are motivated to use Internet pornography in terms of its social value (e.g., to get turned on before sex, to learn new sexual positions, etc.). The fantasy motive refers to using pornography to fantasize about having sex with one of the actors in the video. The mood management motive reflects that people use Internet pornography to feel entertained or to balance their mood (e.g., using pornography when bored/depressed or to relieve sexual frustration). Lastly, the habitual use motive is using pornography as a routine or compulsion. Paul and Shim (2008) posited that using pornography as a mood management motive is positively correlated with habitual use. In other words, using pornography to manage mood can occur habitually as a routine. Similar to this research, Bőthe et al. (2021) constructed a short, reliable measure to assess different pornography use motives. Through this measure, the authors proposed eight motivational factors for pornography use including boredom avoidance, emotional distraction/suppression, and stress reduction.

Research by Esplin et al. (2021) implied that males tend to use pornography to cope with unwanted negative emotions including boredom, loneliness, depression, and anger. The notion of “negative” emotions proposed by Esplin et al. (2021) aligns with the mood management motive for watching pornography proposed by Paul and Shim (2008). Additionally, similar to Paul and Shim (2008), Bőthe et al. (2021) implied that individuals who use pornography to reduce their stress and avoid negative feelings (i.e., emotional distraction/suppression and stress reduction motives) are likely to develop problematic pornography use. Despite the constructs of emotional distraction/suppression and stress reduction being separate motives in Bőthe et al.’s (2021) study, there was a strong correlation (*r* = .779, *p* < .001) between them. Due to this, and because these factors align with the mood management motive for watching pornography proposed by Paul and Shim (2008), we chose to examine the mood management motive over other motives in this study.

## The Current Study

Recent literature examining the motives for watching pornography reveals that specific motives can be more problematic than others (Bőthe et al., 2021; Esplin et al., 2021). It is also suspected that some motives may be more closely associated with sexual aggression in the context of the CM. To date, there have been no studies that have examined the motives for using pornography and their relation to sexual aggression in the context of the CM. Therefore, the current study aims to further examine the relationship between pornography consumption and sexual aggression with respect to the CM by incorporating specific motives for watching pornography, with a distinct focus on the mood management motive. The hypotheses are as follows:

*Hypothesis 1:* HM will be positively associated with sexual aggression.

*Hypothesis 2:* IS will be positively associated with sexual aggression.

*Hypothesis 3:* When testing HM and IS scores in parallel, they will both be positively associated with sexual aggression.

*Hypothesis 4:* Pornography use consumption will not be associated with sexual aggression.

*Hypothesis 5*: Mood management motives for pornography use will moderate the relationship between the primary risk factors of the CM and sexual aggression.

## Method

### Sample

Participants (*N* = 616) were recruited to an online anonymous survey hosted on Qualtrics. A power calculation based on G*Power (Faul et al., 2009) yielded a minimum sample size of *N* = 242 (β = .80, α = .05), suggesting satisfactory power for this study was achieved. To participate, prospective participants^1^1Note that the sample was not exclusive to heterosexual men as having any sexual attraction to women (e.g., being bisexual) still counted as being eligible to participate. had to be male adults aged between 17-25 who were attracted to women and resided in North America at the time of the study. For the analyses, *n* = 160 participants were excluded for completing less than 94% of the survey, *n* = 37 were excluded for not meeting the eligibility requirements, and *n* = 5 were excluded for not consenting to the study, yielding a final sample size *N* = 412 participants.

### Measures

#### Demographics

The demographics section asked participants about their age, ethnicity, and sexual orientation. The Kinsey scale (Kinsey et al., 1948) was used to measure sexual orientation. This scale aims to capture one’s sexual orientation and acknowledges that many individuals are not completely homosexual or completely heterosexual and that sexual attraction is fluid and can fall on a spectrum. This scale was used as participants were not limited to only heterosexual males and needed only to possess some sexual attraction to women (e.g., being bisexual) to be eligible to participate.

#### Perpetration of Sexual Aggression

Participants were presented with two questions related to sexual aggression. The first question asked: “During the last six months, how many times have you kissed, touched, or done anything sexual with another person when that person did not want you to do so?” This question was developed by Ybarra et al. (2011) to describe sexual aggression by using terms that would be comprehensible to children and adolescents. Kohut et al. (2021) used this measure when testing the CM on male adolescents in Croatia, finding test-retest scores to range between *r* = .01 and *r* = .23 in the Zagreb panel and between *r* = .10 and *r* = .30 in the Rijeka panel. Further, because our study focused on measuring sexual aggression in emerging male adults, we added an additional question that replaced “During the last six months” with “From the age of 17”. For the purposes of data analysis, the question that assessed the presence of sexual aggression from the age of 17 was used. For both questions, response options were “Never”, “Once”, and “Several times”. Consistent with Kohut et al. (2021), the sexual aggression variable was dichotomized as “Yes” if someone had perpetrated sexual aggression and “No” if they had not.

#### Hostile Masculinity

HM was assessed by using a 10-item adapted version of the Hostility Toward Women scale, which was initially proposed by Lonsway and Fitzgerald (1995) and used in the Kohut et al. (2021) study. Some examples of items on this scale included “I think that most women would lie just to get ahead” and “I feel that many times women flirt with men just to tease them or hurt them”. Responses were collected on a 5-point scale ranging from “Strongly disagree” to “Strongly agree”, with higher scores suggesting higher levels of HM. This scale is found to be related to other measures of hostility toward women (e.g., Rape Myth Scale, Acceptance of Interpersonal Violence Scale, Adversarial Sexual Beliefs; Lonsway & Fitzgerald, 1995), which supports the scale’s construct validity. Considering its reliability, the Hostility Toward Women scale has obtained coefficients ranging from α = .72-.89 and has produced test-retest scores of *r* = .46 to *r* = .66 (Check et al., 1985; Kohut et al., 2021; Malamuth, 1986).

#### Impersonal Sexuality

Because the definition of IS is unclear in the literature, it has been measured in various ways. In the present study, IS was measured by asking participants the two questions that Malamuth et al. (2000) used to measure sexual promiscuity (which is now considered IS). First, participants were asked how old they were when they first had voluntary sex. Because this study is testing the CM on a targeted age range, participants had to choose an age ranging from 12 to 25 as the age they became sexually active. They were also given the option of “Not applicable” for those who have never had intercourse and “Other” for those who had sex younger than age 12. For participants who indicated never having intercourse (32.8%, *n* = 135), their current age was entered for this variable. This approach was employed in previous studies (Baer et al., 2015; Malamuth et al., 2000; Malamuth et al., 2012). Second, participants were asked to identify how many sexual partners they had since the age of 14 by providing a numeric amount. IS scores were considered high if participants indicated their first voluntary sexual experience at an early age (e.g., earlier than the average age for all participants) and if participants had a number of sexual partners greater than the mean for all participants. However, because two distinct questions were asked (age of first intercourse and the number of sexual partners since the age of 14), we tested each component individually.

#### Pornography Consumption

In the current study, pornography was defined as “A material (e.g., text, picture, video) that creates or elicits sexual feelings or thoughts and contains explicit exposure or descriptions of sexual acts involving the genitals, such as vaginal or anal intercourse, oral sex, or masturbation.” (Bőthe et al., 2021, p. 185). Similar definitions of pornography have been used in previous studies (Baer et al., 2015; Dawson et al., 2019; Kohut et al., 2021). In the current study, pornography consumption was measured by asking two questions. First, we asked participants “During the last six months, how often have you used pornography”. Second, because participants were emerging male adults, we asked “From the age of 17, how often on average have you used pornography”. Responses were collected on an 8-point scale ranging from “Not once” to “Several times a day’’, with higher scores suggesting greater pornography consumption. Stability coefficients for similar questions have been shown to range between *r* = .55 and *r* = .75 (Kohut et al., 2021).

#### Pornography Use Motivation

Pornography use motivation was measured using the Pornography Use Motivations Scale (PUMS; Bőthe et al., 2021). Bőthe and colleagues (2021) consider eight motivational factors for using pornography: Sexual Pleasure (e.g., “I watch porn to arouse myself”), Sexual Curiosity (e.g., “I watch porn to become better in bed”), Fantasy (e.g., “I watch porn because I can be a part of things that I cannot experience in real life”), Boredom Avoidance (e.g., “I watch porn because I am bored”), Lack of Sexual Satisfaction (e.g., “I watch porn because I miss sex”), Emotional Distraction or Suppression (e.g., “I watch porn to distract myself from my negative thoughts”), Stress Reduction (e.g., “I watch porn because it calms me down”), and Self-Exploration (e.g., “I watch porn to get to know my own sexual desires better”). Responses were collected on a 7-point scale which ranged from “Never” to “All the time”, with higher responses indicating greater endorsement for that motivation. In the present study, only mood management motives (i.e., emotional distraction/suppression and stress reduction) were considered and were incorporated as one subscale. PUMS produced strong psychometric properties when considering its reliability and construct validity; however, the scale was only measured using a sample of university students (Bőthe et al., 2021).

### Procedure

Participants were recruited for this study through a recruitment poster posted on social media platforms (i.e., Reddit, Facebook, Twitter, Instagram) and by word-of-mouth. Interested participants were invited to learn more about the study by clicking on the survey link, at which point they were directed to an informed consent form describing the study in more detail. If they provided their consent, participants completed an anonymous online survey on Qualtrics. Because stigma plays an important role in pornography use (Kohut et al., 2020), an anonymous survey format was used to encourage truthful responding. The survey first asked participants demographic questions followed by whether they had engaged in sexual activity with someone who did not want to. Next, participants were asked about their own hostile attitudes towards women, their experiences with impersonal sexuality, about their pornography consumption habits, and about their motivations behind using pornography. The entire survey took approximately 10 minutes to complete. All study procedures were cleared by the Saint Mary’s University Research Ethics Board (REB # 22-008).

### Data Analysis Plan

First, three binary logistic regressions were conducted to assess the unique relationships between sexual aggression and HM, IS, and pornography consumption. Next, a multiple logistic regression analysis was used to assess whether HM and IS together were associated with sexual aggression. Lastly, a moderation analysis was conducted in PROCESS to determine whether the mood management factor of the PUMS moderated the relationships between the combined HM and IS scores and sexual aggression. As in previous studies (Malamuth et al., 2000; Malamuth et al., 2012; Vega & Malamuth, 2007), a single risk score was calculated based on the confluence of the key composite predictors. First, we divided the HM and IS scores separately into one of the three different categories (i.e., 1 = low risk, 2 = medium risk, 3 = high risk). Participants who scored in the lowest 25% of the distribution were assigned a “1”, those who scored in the middle 50% were assigned a “2”, and those who scored in the highest 25% of the distribution were assigned a “3”. We then combined both individual risk scores together to obtain a total composite risk score (i.e., 1-2 = low risk, 3-4 = medium risk, 5-6 = high risk). In the moderation model, the composite risk score was the predictor, sexual aggression was the outcome, and scores on the mood management motive of the PUMS was the moderator. If a significant moderation effect was detected, it was planned that a combination of the pick-a-point approach and the Johnson-Neyman procedure would be used to deconstruct the effect (Field, 2017).

## Results

### Descriptive Statistics

Descriptive statistics and intercorrelations for key study variables are reported in Table 1, with additional demographic information presented in Table 2. The mean age of the sample was 20.96 years (*SD* = 2.72). In terms of sexual aggression, 19.8% (*n* = 81) of participants reported they were sexually aggressive since the age of 17.

**Table 1:** Descriptive Statistics and Intercorrelations for Key Study Variables in the Current Study

Variable	*M*	*SD*	Correlation				
			1	2	3	4	5
1. Hostile masculinity	21.60	6.45	(.78)				
2. Age at first sexual experience	18.37	3.17	.06	–			
3. Number of sexual partners	4.45	10.17	.08	.26***	–		
4. Pornography use motives	16.10	9.23	.15**	.06	-.05	(.93)	
5. Pornography consumption	5.37	1.82	-.07	.02	-.06	.24***	–
6. Sexual aggression	–	–	.13*	.13*	.21***	.13*	-.08

*Note*. Impersonal sexuality is measured through both age at first sexual experience and number of sexual partners. Alpha coefficients for all scales are presented on the principal diagonal (where applicable). *N* = 160.

*p < .05. **p < .01. ***p < .001.

**Table 2:** Demographic Information for the Study Sample

Variable	*N*	%
Ethnicity (*n* = 420)		
White (e.g., Caucasian, European Descent)	288	69.9
Chinese	11	2.7
South Asian (e.g., East Asian, Sri Lankan, Pakistani)	18	4.4
Black (e.g., African, Haitian, Jamaican)	42	10.2
Filipino	6	1.5
Latin American (e.g., Mexican, South/Central America)	25	6.1
Aboriginal (e.g., North American Indian, Metis, Inuit, First Nations)	7	1.7
Southeast Asian (e.g., Vietnamese, Cambodian, Malaysian, Laotian)	10	2.4
Middle Eastern	30	7.3
West Asian (e.g., Iranian, Afghan)	4	1
Japanese	6	1.5
Korean	2	0.5
Not sure	3	0.7
Ethnicity not listed	16	3.9
Sexual orientation (*n* = 405)		
Heterosexual (straight)	270	66.7
Mostly heterosexual (mostly straight)	66	16.3
Bisexual	48	11.9
Mostly homosexual (mostly gay/lesbian)	3	0.7
Homosexual (gay/lesbian)	2	0.5
Asexual	2	0.5
Other	11	2.7
I do not wish to answer this question	3	0.7

*Note*. Participants could select multiple ethnicities. *N* = 412.

### Association Between HM, IS, Pornography Consumption, and Sexual Aggression

Table 3 presents the results of the binary logistic regressions. Support for Hypothesis 1 was generated in that higher HM scores were associated with greater perpetration of sexual aggression (Nagelkerke *R*^2^ = .03). There was no significant effect when testing the first component of IS (age of first intercourse) and sexual aggression (Nagelkerke *R*^2^ = .01). However, there was a significant finding for the second component of IS (the number of sexual partners since the age of 14). In support of Hypothesis 2, higher IS scores (higher number of sexual partners since the age of 14) were associated with greater perpetration of sexual aggression (Nagelkerke *R*^2^ = .06). Due to this component of the IS being significant, we chose the number of sexual partners since the age of 14 to represent the IS measure for the remainder of the analyses. As expected, and in support of Hypothesis 4, no relationship was identified between pornography consumption and sexual aggression (Nagelkerke *R*^2^ = .00). A fourth post-hoc binary logistic regression was conducted to examine whether using pornography for mood management was associated with sexual aggression. Interestingly, we found a significant and positive relationship between using pornography for mood management and sexual aggression (Nagelkerke *R*^2^ = .03).

**Table 3:** Logistic Regression Results for the Associations Between HM, IS, Pornography Consumption, Mood Management Motive for Pornography Use, and Sexual Aggression

Variable	*B* (*SE*)	*OR* [95% CI]	χ^2^
Binary logistic regression			
HM	.05 (.02)**	1.05 [1, 1.09]	7.49**
IS (age of first intercourse)	-.04 (.04)	0.96 [0.89, 1.03]	1.20
IS (number of sexual partners)	.05 (.02)***	1.05 [1.02, 1.08]	14.65***
Pornography consumption	-.05 (.07)	0.95 [0.83, 1.08]	0.62
Multiple logistic regression			
HM	.05 (.02)***	1.05 [1.01, 1.09]	19.67***
IS (number of sexual partners)	.05 (.02)***	1.05 [1.02, 1.08]	
Post Hoc Test			
Mood management motive for pornography use	.04 (.01)**	1.04 [1.01, 1.06]	6.84**

*Note. B =* standardized regression coefficient.

*p < .05. **p < .01. ***p < .001.

### Association Between HM, IS, Pornography Use Motives, and Sexual Aggression

Table 3 presents the results of the multiple logistic regression analysis. As anticipated, and in line with Hypothesis 3, both HM and IS (number of sexual partners) remained significantly associated with sexual aggression when entered simultaneously into the analysis (Nagelkerke *R*^2^ = .09). Table 4 presents the results of the moderation analysis. This analysis was conducted to test whether the mood management motive for pornography use would moderate the association between the composite risk score (HM and IS combined) and sexual aggression. After conducting the moderation analysis, contrary to Hypothesis 5, we found no significant moderation effect. Due to a lack of statistical significance, we could not proceed with the pick-a-point approach or the Johnson-Neyman procedure.

**Table 4:** Moderation Analysis Between Composite Risk Scores, Mood Management Motive for Pornography Use, and Sexual Aggression

Variable	*b*	*SE*	*z*	*p*	95% Cl
Composite risk scores (HM + IS)	0.25	0.13	2.01	.045	[0.01, 0.5]
Mood management motive	0.02	0.04	0.54	.592	[-0.06, 0.1]
Composite risk scores x mood management motive	.003	0.01	0.42	.673	[-0.01, -0.02]

*Note*. *b* = unstandardized regression coefficient.

## Discussion

The current study examined the association between the primary risk factors of the CM with a focus on the role of pornography use as a secondary risk factor for sexual aggression. This is the first study to test the role of pornography use motives as a secondary risk factor in the CM, and to use a reliable and valid measure of pornography use (PUMS; Bőthe et al., 2021) instead of a single-item question. In summary, there was support generated for the core components of the CM and their influence on sexually aggressive behaviour, supporting our first three hypotheses; however, the association between pornography and sexual aggression was more complicated.

Consistent with previous findings testing the CM (Malamuth, 1986; Malamuth, 2018; Malamuth et al., 1995; Malamuth et al., 2021), the results of our study revealed an association between the primary key factors of the CM and sexual aggression. As previously discussed, there is an inconsistency with the construct of IS and how it is measured. The current study attempted to follow previous study methods of measuring IS by examining participants’ first age of intercourse and number of sexual partners since the age of 14 (Kohut et al., 2021; Malamuth et al., 1995; Malamuth et al., 2000). Despite measuring IS in two ways, we only found a significant association between the number of sexual partners since the age of 14 and sexual aggression. One problem with asking the participants their age of first intercourse was the lack of specificity as it could have led to participants excluding specific sexual experiences (e.g., oral/anal sex). There are different approaches to assess the measure of IS across studies; however, there is still not a proper assessment of IS. (Malamuth et al., 1991; Malamuth et al., 1996; Malamuth et al., 2021; Vega & Malamuth, 2007). Despite this, the findings of this study suggest that the primary risk factors for men’s perpetration of sexual aggression against women as proposed by Malamuth (1986) are still relevant to understanding risk almost forty years later.

Previous research findings (Huntington et al., 2020; Malamuth et al., 2000; Vega & Malamuth, 2007) stress that secondary risk factors (e.g., pornography consumption) enhance the prediction of sexual aggression in the presence of primary risk factors. Consistent with our fourth hypothesis, our findings suggested that general pornography consumption had no independent relationship with sexual aggression. This finding is consistent with previous literature suggesting that pornography may reinforce the tendency to be sexually aggressive only if men are already considered high-risk due to the presence of the primary risk factors (Baer et al., 2015; Huntington et al., 2020; Kingston et al., 2009; Malamuth, 2018; Malamuth et al., 2000; Vega & Malamuth, 2007).

Interestingly, a post-hoc analysis revealed an independent association between watching pornography as a mood management motive and sexual aggression. This was an interesting finding as both Paul and Shim (2008) and Esplin et al. (2021) have indicated that pornography use is mostly related to managing one’s emotions. This study builds on the literature by suggesting that using pornography as a mood management tool may be an independent risk factor for sexual aggression. Despite this finding, it is important to note that the use of pornography as a mood management motive did not moderate the association between the primary risk factors of the CM and sexual aggression. Thus, we found no support for our fifth hypothesis, indicating only partial support for considering pornography consumption as a secondary risk factor in the CM.

Overall, our results suggest that it is not the use of pornography that is associated with sexual aggression, but instead the motive behind watching pornography. This is in spite of past research suggesting that pornography use could increase the risk of sexual aggression in men who score high on the primary risk factors (Malamuth, 2018; Malamuth et al., 2000; Vega & Malamuth, 2007). Recent research testing the CM has revealed mixed or non-significant results concerning the relationship between pornography use, the primary risk factors, and sexual aggression, ultimately calling the utility of pornography as a secondary risk factor into question (Huntington et al., 2020; Kohut et al., 2021; Malamuth et al., 2021; Wright et al., 2021). More research is needed to resolve these inconsistencies.

Relevant to this study, Kohut et al. (2020) highlighted that pornography use is usually measured with one-item assessments that adopt terminologies with different meanings (e.g., pornography, erotica, smut, etc.). It is important to acknowledge that the CM was developed several decades ago when the construct of pornography was assessed by the consumption of erotic magazines (e.g., Playboy, Penthouse, etc.) (Malamuth, 1998; Malamuth et al., 2000; Vega & Malamuth, 2007). Therefore, previous studies examining pornography’s role in the CM involved films or books and did not include Internet pornography. Because the core constructs of the CM were developed decades ago when pornography was different, this may limit the theoretical explanation that pornography can add to the CM.

In this study, pornography consumption was measured as a single-item question and was not associated with sexual aggression. Kohut et al. (2020) suggested that the usage of properly validated instruments is most beneficial to understanding the construct of pornography in research. When using a validated instrument to assess pornography use motives (PUMS; Bőthe et al., 2021), we found an association between the mood management motive and sexual aggression. These findings imply that the way pornography is assessed matters. Research regarding pornography may still result in inconsistent conclusions as the term “pornography” is being utilized to describe various constructs (Kohut et al., 2020). As such, there is a need to develop both specific and standardized measurements of pornography consumption to circumvent these challenges.

### Study Implications

This study has provided a first look at the role that pornography use motives may play in sexual aggression within the core constructs of the CM. Further, our results have provided additional evidence to support the notion that there is an association between HM and IS and sexual aggression. While our study demonstrated that specific pornography use motives and not just general pornography consumption are related to sexual aggression, future research is needed to replicate this finding. Additionally, the findings from the present study imply that the two core constructs of the CM are still relevant in predicting sexual aggression in men today and should be assessed by clinicians who work with men who may be at risk for perpetrating sexual aggression. If researchers continue to find inconsistent results regarding the role of pornography in the CM while using valid tools to measure pornography, it may suggest a need to reconsider whether pornography is truly a secondary risk factor in the CM.

The independent association identified between the mood management motive for using pornography and sexual aggression raises intriguing implications. This finding is noteworthy as it suggests that consuming pornography as a mood management tool may be a risk factor for sexual aggression independent of other risk factors. When considering the wider literature, there is an association between coping using sex and sexual aggression. For instance, in their development of the Coping Using Sex Inventory, Cortoni and Marshall (2001) suggested that individuals who have sexually offended consistently report engaging in sexual activity (e.g., fantasies, masturbation, pornography, or sex with a partner) as a coping strategy to deal with stressful circumstances. Given the similarities between using pornography as a mood management motive and using sex to cope (as per the Coping Using Sex Inventory), this finding becomes less surprising. Taken together, the findings from this study imply that using sex as a coping strategy, particularly pornography consumption for mood management, seems to be a characteristic of individuals who have sexually offended. As such, it is important to consider specific motives for watching pornography when attempting to understand sexual aggression in men who consume pornography. Given the novelty of this finding, it merits further scholarly attention.

The findings of this research can be beneficial for clinicians and therapists. As discussed, research by Esplin et al. (2021) examining the motives for pornography use suggested that clinicians who work with pornography users should understand the motives that their client has for watching pornography to inhibit problematic pornography consumption. The term “problematic pornography consumption” refers to “pornography that leads to significant negative interpersonal or personal consequences for the use” (Sniewski et al., 2018, p. 217). Moreover, Bőthe et al. (2021) suggested that individuals may use pornography to reduce their negative feelings, and this can impact the severity of one’s problematic pornography consumption. Nevertheless, clinicians are still concerned when it comes to helping their clients manage their problematic pornography consumption. Thus, in addition to assessing the primary risk factors for sexual aggression (i.e., HM and IS), clinicians should pay attention to their clients’ pornography use motives as well. Specifically, if their clients are using pornography for mood management, the need for intervention is amplified. Intervention can allow their clients to understand the motives behind their pornography consumption, recognize certain triggers, and find healthier coping alternatives. This research is a starting point for further research into the role that specific pornography use motives play in the CM and how clinicians can use this information to prevent sexual aggression.

### Future Research Directions

There continues to be a gap in the literature given the age of the CM and how it has not been reassessed against social changes and changes in the scientific literature. For example, dating norms have changed over time and with the evolution of technology (e.g., the introduction of dating apps), which could influence how the components of CM should be measured (particularly IS). Future research should aim to find a proper tool with strong psychometric properties that also acknowledges societal changes and how they may have impacted the components of the CM. Furthermore, our analyses considered only general pornography consumption and specific motives for pornography use. While this approach measured other aspects of pornography, it does not address the role violent pornography plays in the CM. Past research has suggested that men high in the primary risk factors have a high likelihood of exposing themselves to violent pornography as they may be attracted to imagery that reinforces their impersonal and hostile orientation to sexuality, which can in turn aggravate their sexually aggression tendencies (Malamuth, 2018; Malamuth et al., 2000; Malamuth et al., 2012). Baer et al. (2015) highlighted that violent pornography can reinforce or normalize risk factors for sexual aggression through viewing degrading or dominating scenes. Thus, subsequent research should also consider aspects of pornography other than consumption (e.g., the consumption medium, different genres, and contents, etc.), along with how this might impact the association between the primary risk factors of the CM and sexual aggression. An additional consideration for future research is to not limit the measure of sexual aggression to only physical acts of sexual aggression given the previously identified strong associations between pornography use and verbal sexual aggression as compared to physical sexual aggression (Wright et al., 2016).

Lastly, despite the many strengths of various studies testing the CM, many researchers have not paid attention to other secondary risk factors that have been associated with sexual aggression perpetration. For instance, Abbey et al. (2011) have suggested that delinquency and misperceptions of sexual intent are related to sexual aggression. Further, Baer et al. (2015) and Wright et al. (2021) also implied that sex drive may play a role in the relationship between pornography use and sexual aggression. Thus, future research should also consider additional secondary risk factors when exploring ways to expand the CM.

### Limitations

The present study does not come without limitations. First, the sample was limited to North American men who identified primarily as White. As there may be cross-cultural differences in the core components of the CM, future research should aim to recruit a more diverse sample. A second limitation is that we relied on a self-reported measure of sexual aggression. It is possible that some participants responded in a socially desirable manner and may therefore not have answered truthfully. A third limitation is the single-item question to measure pornography use. Kohut et al. (2020) suggest that while the term “pornography use” refers to voluntarily self-exposure to pornographic materials, individuals can also be exposed unwillingly to pornography. Additionally, they may use pornography individually or with someone else, as well as both privately and publicly. Because there are multiple ways to conceptualize “pornography use”, research measuring pornography consumption can produce inconsistent results. Given the fact that our study produced contradicting results to past studies, there is a need to develop a standardized measure of pornography consumption. A final limitation is that this study employed a cross-sectional design which prevented us from examining the core constructs of the CM and pornography usage over time. Though it is useful to capture this data at a specific point in time, it can be assumed that the characteristics represented in the CM could have developed after the acts of sexual aggression; therefore, the conclusion that the primary risk factors contributed to the sexual aggression reported in this study cannot be drawn. Longitudinal studies are needed to identify the chronological sequencing and temporal order of the primary risk factors for sexual aggression proposed by the CM and to determine whether the CM can predict sexual aggression.

### Conclusion

The findings from this study support the core components of the CM and suggest that there is an association between the primary risk factors (HM, IS) and sexual aggression. Moreover, although pornography consumption is unrelated to sexual aggression, there appears to be an association between using pornography for mood management and sexual aggression. Despite this, using pornography for mood management does not moderate the relationship between the risk factors in the CM and sexual aggression. Although the relationship between pornography use, sexual aggression, and the core components of the CM remains uncertain, this study provides support for considering secondary risk factors as a way to expand the CM.

## References

[r1] Abbey, A., Jacques-Tiura, A. J., & LeBreton, J. M. (2011). Risk factors for sexual aggression in young men: An expansion of the Confluence Model. Aggressive Behavior, 37(5), 450–464. 10.1002/ab.20399 21678429 PMC3139770

[r2] Baer, J. L., Kohut, T., & Fisher, W. A. (2015). Is pornography use associated with anti-woman sexual aggression? Re-examining the Confluence Model with third variable considerations. The Canadian Journal of Human Sexuality, 24(2), 160–173. 10.3138/cjhs.242-A6

[r3] Bauserman, R. (1996). Sexual aggression and pornography: A review of correlational research. Basic and Applied Social Psychology, 18(4), 405–427. 10.1207/s15324834basp1804_4

[r4] Bőthe, B., Tóth-Király, I., Bella, N., Potenza, M. N., Demetrovics, Z., & Orosz, G. (2021). Why do people watch pornography? The motivational basis of pornography use. Psychology of Addictive Behaviors, 35(2), 172–186. 10.1037/adb0000603 32730047

[r5] Check, J. V. P., Malamuth, N. M., Elias, B., & Barton, S. (1985). On hostile ground. Psychology Today, 19(4), 56–61.

[r6] Cortoni, F., & Marshall, W. L. (2001). Sex as a coping strategy and its relationship to juvenile sexual history and intimacy in sexual offenders. Sexual Abuse, 13(1), 27–43. 10.1177/107906320101300104

[r7] Dawson, K., Tafro, A., & Štulhofer, A. (2019). Adolescent sexual aggressiveness and pornography use: A longitudinal assessment. Aggressive Behavior, 45(6), 587–597. 10.1002/ab.21854 31432547

[r8] Esplin, C. R., Hatch, S. G., Hatch, H. D., Deichman, C. L., & Braithwaite, S. R. (2021). What motives drive pornography use? The Family Journal, 29(2), 161–174. 10.1177/1066480720956640

[r9] Faul, F., Erdfelder, E., Buchner, A., & Lang, A.-G. (2009). Statistical power analyses using G*Power 3.1: Tests for correlation and regression analyses. Behavior Research Methods, 41(4), 1149–1160. 10.3758/BRM.41.4.1149 19897823

[r10] Ferguson, C. J., & Hartley, R. D. (2022). Pornography and sexual aggression: Can meta-analysis find a link? Trauma, Violence, & Abuse, 23(1), 278–287. 10.1177/1524838020942754 32691692

[r11] Field, A. (2017). *Discovering Statistics Using IBM SPSS Statistics* (5th ed.). SAGE.

[r12] Huntington, C., Pearlman, D. N., & Orchowski, L. (2020). The Confluence Model of Sexual Aggression: An application with adolescent males. Journal of Interpersonal Violence, 37(1-2), 623–643. 10.1177/0886260520915550 32306817

[r13] Kingston, D. A., Fedoroff, P., Firestone, P., Curry, S., & Bradford, J. M. (2008). Pornography use and sexual aggression: The impact of frequency and type of pornography use on recidivism among sexual offenders. Aggressive Behavior, 34(4), 341–351. 10.1002/ab.20250 18307171

[r14] Kingston, D. A., Malamuth, N. M., Fedoroff, P., & Marshall, W. L. (2009). The importance of individual differences in pornography use: Theoretical perspectives and implications for treating sexual offenders. Journal of Sex Research, 46(2–3), 216–232. 10.1080/00224490902747701 19308844

[r15] Kinsey, A. C., Pomeroy, W. B., & Martin, C. E. (1948). *Sexual behavior in the human male.* Saunders. 10.2105/ajph.93.6.894 PMC1447861 12773346

[r16] Kohut, T., Balzarini, R. N., Fisher, W. A., Grubbs, J. B., Campbell, L., & Prause, N. (2020). Surveying pornography use: A shaky science resting on poor measurement foundations. Journal of Sex Research, 57(6), 722–742. 10.1080/00224499.2019.1695244 31821049

[r17] Kohut, T., Landripet, I., & Štulhofer, A. (2021). Testing the Confluence Model of the association between pornography use and male sexual aggression: A longitudinal assessment in two independent adolescent samples from Croatia. Archives of Sexual Behavior, 50(2), 647–665. 10.1007/s10508-020-01824-6 33083941

[r18] Lonsway, K. A., & Fitzgerald, L. F. (1995). Attitudinal antecedents of rape myth acceptance: A theoretical and empirical reexamination. Journal of Personality and Social Psychology, 68(4), 704–711. 10.1037/0022-3514.68.4.704

[r19] Malamuth, N. M. (1986). Predictors of naturalistic sexual aggression. Journal of Personality and Social Psychology, 50(5), 953–962. 10.1037/0022-3514.50.5.953 3712232

[r20] Malamuth, N. M. (1996). The Confluence Model of Sexual Aggression: Feminist and evolutionary perspectives. In D. M. Buss & N. M. Malamuth (Eds.), *Sex, power, conflict: Evolutionary and feminist perspectives* (pp. 269–295). Oxford University Press.

[r21] Malamuth, N. M. (1998). The Confluence Model as an organizing framework for research on sexually aggressive men. In R. G. Geen & E. Donnerstein (Eds.), *Human aggression: Theories, research, and implications for social policy* (pp. 229–245). Elsevier. 10.1016/B978-012278805-5/50010-9

[r22] Malamuth, N. M. (2018). “Adding fuel to the fire”? Does exposure to non-consenting adult or to child pornography increase risk of sexual aggression? Aggression and Violent Behavior, 41, 74–89. 10.1016/j.avb.2018.02.013

[r23] Malamuth, N. M., Addison, T., & Koss, M. P. (2000). Pornography and sexual aggression: Are there reliable effects and can we understand them? Annual Review of Sex Research, 11, 26–91. 10.1080/10532528.2000.10559784 11351835

[r24] Malamuth, N. M., & Hald, G. M. (2016). The confluence mediational model of sexual aggression. In D. P. Boer (Ed.), *The Wiley handbook on the theories, assessment and treatment of sexual offending* (Vol. 1, pp. 53–71). John Wiley & Sons. 10.1002/9781118574003.wattso003

[r25] Malamuth, N. M., Hald, G. M., & Koss, M. P. (2012). Pornography, individual differences in risk and men’s acceptance of violence against women in a representative sample. Sex Roles, 66(7–8), 427–439. 10.1007/s11199-011-0082-6

[r26] Malamuth, N. M., Heavey, C. L., & Linz, D. (1996). The Confluence Model of Sexual Aggression: Combining hostile masculinity and impersonal sex. Journal of Offender Rehabilitation, 23(3–4), 13–37. 10.1300/J076v23n03_03

[r27] Malamuth, N. M., Lamade, R. V., Koss, M. P., Lopez, E., Seaman, C., & Prentky, R. (2021). Factors predictive of sexual violence: Testing the four pillars of the Confluence Model in a large diverse sample of college men. Aggressive Behavior, 47(4), 405–420. 10.1002/ab.21960 33719096

[r28] Malamuth, N. M., Linz, D., Heavey, C. L., Barnes, G., & Acker, M. (1995). Using the Confluence Model of Sexual Aggression to predict men’s conflict with women: A 10-year follow-up study. Journal of Personality and Social Psychology, 69(2), 353–369. 10.1037/0022-3514.69.2.353 7643309

[r29] Malamuth, N. M., Sockloskie, R. J., Koss, M. P., & Tanaka, J. S. (1991). Characteristics of aggressors against women: Testing a model using a national sample of college students. Journal of Consulting and Clinical Psychology, 59(5), 670–681. 10.1037/0022-006X.59.5.670 1955602

[r30] Paul, B., & Shim, J. W. (2008). Gender, sexual affect, and motivations for internet pornography use. International Journal of Sexual Health, 20(3), 187–199. 10.1080/19317610802240154

[r31] Sniewski, L., Farvid, P., & Carter, P. (2018). The assessment and treatment of adult heterosexual men with self-perceived problematic pornography use: A review. Addictive Behaviors, 77, 217–224. 10.1016/j.addbeh.2017.10.010 29069616

[r32] Vega, V., & Malamuth, N. M. (2007). Predicting sexual aggression: The role of pornography in the context of general and specific risk factors. Aggressive Behavior, 33(2), 104–117. 10.1002/ab.20172 17441011

[r33] Wright, P. J., Paul, B., & Herbenick, D. (2021). Pornography, impersonal sex, and sexual aggression: A test of the Confluence Model in a national probability sample of men in the U.S. Aggressive Behavior, 47(5), 593–602. 10.1002/ab.21978 34076267

[r34] Wright, P. J., Tokunaga, R. S., & Kraus, A. (2016). A meta-analysis of pornography consumption and actual acts of sexual aggression in general population studies: Pornography and sexual aggression. Journal of Communication, 66(1), 183–205. 10.1111/jcom.12201

[r35] Yang, D. O., & Youn, G. (2012). Effects of exposure to pornography on male aggressive behavioral tendencies. The Open Psychology Journal, 5, 1–10. 10.2174/1874350101205010001

[r36] Ybarra, M. L., Mitchell, K. J., Hamburger, M., Diener-West, M., & Leaf, P. J. (2011). X-rated material and perpetration of sexually aggressive behavior among children and adolescents: Is there a link? Aggressive Behavior, 37(1), 1–18. 10.1002/ab.20367 21046607

